# Supplementary material for: Comparative genomics provides new insights into the diversity, physiology, and sexuality of the only industrially exploited tremellomycete: Phaffia rhodozyma
Source: BMC Genomics. 2016 Nov 9;17:901. doi: 10.1186/s12864-016-3244-7 (PMC5103461; doi:10.1186/s12864-016-3244-7)
Supplement: Additional file 6: — List of orphan genes with links to PFAM (related to Additional file 1: Table S1). (ZIP 1428 kb) [file 12864_2016_3244_MOESM6_ESM.zip › PFAM_UNIQUE/G02353_P.html]

#                                                                            --- full sequence --- -------------- this domain -------------   hmm coord   ali coord   env coord
  
# target name        accession   tlen query name           accession   qlen   E-value  score  bias   #  of  c-Evalue  i-Evalue  score  bias  from    to  from    to  from    to  acc description of target
  
#------------------- ---------- ----- -------------------- ---------- ----- --------- ------ ----- --- --- --------- --------- ------ ----- ----- ----- ----- ----- ----- ----- ---- ---------------------
  
G02353\_P             -            282 DUF566               PF04484.7    313       6.2    4.9   9.5   1   1     0.031       8.2    4.5   9.5     6    87    61   144    40   185 0.56 - Family of unknown function (DUF566)  
G02353\_P             -            282 DUF605               PF04652.11   380       8.9    4.2  10.8   1   1      0.18        11    3.9  10.8   211   324    56   157    35   211 0.47 - Vta1 like  
G02353\_P             -            282 Speriolin\_N          PF15058.1    200       8.6    4.9   8.3   1   1    0.0044        13    4.3   8.3    43   153    65   141    42   175 0.58 - Speriolin N terminus
